# Biological effects of combinations of structurally diverse human milk oligosaccharides

**DOI:** 10.3389/fped.2024.1439612

**Published:** 2024-11-05

**Authors:** Anita Wichmann

**Affiliations:** Global Regulatory Affairs HMOs, Early Life & Medical Nutrition, DSM-Firmenich, Hørsholm, Denmark

**Keywords:** human milk oligosaccharides, pediatric nutrition, gut microbiota, pathogen inhibition, cognitive development

## Abstract

Human milk oligosaccharides (HMOs) are a diverse group of structures and an abundant bioactive component of breastmilk that contribute to infant health and development. Preclinical studies indicate roles for HMOs in shaping the infant gut microbiota, inhibiting pathogens, modulating the immune system, and influencing cognitive development. In the past decade, several industrially produced HMOs have become available to fortify infant formula. Clinical intervention trials with manufactured HMOs have begun to corroborate some of the physiological effects reported in preclinical studies, especially modulation of the gut microbiota in the direction of breastfed infants. As more HMOs become commercially available and as HMOs have some shared mechanisms of action, there is a need to better understand the unique and differential effects of individual HMOs and the benefits of combining multiple HMOs. This review focuses on the differential effects of different HMO structural classes and individual structures and presents a scientific rationale for why combining multiple structurally diverse HMOs is expected to exert greater biological effects.

## 1 Introduction

The associations between breastfeeding, intestinal bacteria composition, and improved infant health and survival were recognized already in the late 19th century. Pioneering work by many scientists and physicians in the early 20th century led to the discovery and characterization of the “bifidus factor” in breastmilk as the carbohydrate fraction composed of human milk oligosaccharides (HMOs) (1). Today, more than 200 molecular species of HMOs have been detected (2), and the structures of more than 160 HMOs have been elucidated (3). Although many different HMO structures are present in breastmilk, the 10 most abundant HMOs have been reported to account for more than 70% of the total HMO concentration (4). The role of HMOs in shaping the infant gut microbiota has been well-established, and evidence also indicates roles for HMOs in pathogen inhibition, immune modulation and cognitive development (5–7).

The benefits of breastfeeding on growth, immunity, cognition, and other functions important for optimal development are substantiated by an extensive body of scientific research (8–11). The World Health Organization recommends exclusive breastfeeding during the first six months of life and continued breastfeeding for up to two years or beyond (12). However, due to a variety of social, economic and medical factors, fewer than half of infants under 6 months old are exclusively breastfed (12). The only suitable alternative to breastmilk that meets the nutritional requirements of infants is infant formula, which aims to resemble the composition and functionality of breastmilk as closely as possible (13). As HMOs are the third most abundant solid component of breastmilk (14), the HMO fraction represents the largest compositional gap between breastmilk and infant formula.

Despite awareness of the compositional and physiological importance of HMOs in breastmilk, HMOs could not be produced efficiently at large scale during the 20th century. To mimic the bifidogenic effect of HMOs, other oligosaccharides such as galacto-oligosaccharides (GOS), fructo-oligosaccharides (FOS) and polydextrose (PDX) were introduced to infant formula (15) and are still commonly found in infant formula products today. However, these oligosaccharides are not present at appreciable levels (GOS) or at all (FOS, PDX) in human milk, and they are structurally distinct from HMOs, lacking the monosaccharide units present in HMOs such as fucose, N-acetylglucosamine (GlcNAc) and *N*-acetyl-D-neuraminic acid (Neu5Ac; also known as sialic acid).

Only in the past decade have technological developments made it possible to produce HMOs at large scale and to fortify infant formula with HMOs. The first HMOs to receive regulatory approvals were 2′-fucosyllactose (2′-FL) and lacto-*N*-neotetraose (LNnT), which received approvals in the U.S. in September and October 2015 and in the EU in March 2016 (16). Infant formula products fortified with 2′-FL or the combination of 2′-FL and LNnT were introduced to the market soon thereafter. Currently, there are regulatory approvals for eight HMOs in the U.S., EU and UK and five to six HMOs in many jurisdictions worldwide such as Australia/New Zealand, Brazil, Thailand and Singapore. In 2023, 2′-FL and LNnT received the first regulatory approvals for HMOs in China. Oral supplementation with manufactured HMOs has been evaluated in at least 26 clinical trials (17). The majority of HMO intervention trials have been conducted in infants, but HMO supplementation has been evaluated in children and adults as well. All these clinical trials reported that consumption of manufactured HMOs was safe and well-tolerated, and many of the trials in infants also reported physiological effects of HMOs, including gut microbiota composition and stooling patterns more similar to breastfed infants and reduced incidence of infections (17).

Despite the progress in HMO production capabilities, clinical evidence and regulatory approvals, infant formula products fortified with HMOs represent a small fraction of the infant formula market today. Although a few infant formula manufacturers have launched products with five or six HMOs, currently most infant formula brands that do contain HMOs are fortified with only one or two. As the number of HMOs that can be produced large-scale increases, there is also increased scrutiny regarding what benefits each additional HMO can provide. This review aims to present evidence regarding the biological effects and potential health benefits of combining multiple HMOs with diverse structures.

## 2 Expected benefits of combining multiple HMOs

Due to the complex logistics and sample sizes necessary for conducting clinical trials in young infants, it is not feasible to compare the physiological effects of many individual HMOs and combinatorial mixtures of HMOs simultaneously in a clinical trial. Hence, preclinical studies provide much of the knowledge on the mechanisms by which different HMOs may exert effects on gut microbiota composition, pathogen inhibition and cognitive development. Although HMOs have many overlapping and redundant effects, there is some evidence for unique and differential benefits of different structural classes of HMOs and different individual HMO structures. Here, the rationale and scientific evidence are presented for why supplementation with multiple HMOs is expected to bring greater health benefits to infants and young children compared to addition of only one or two HMOs, namely:

1. Microbial degradation of HMOs from the three major structural classes (fucosylated, neutral core, sialylated) releases three different molecules: fucose, N-acetylglucosamine (GlcNAc) and *N*-acetyl-D-neuraminic acid (Neu5Ac; also known as sialic acid), which have unique structures and biological functions. These HMO-derived monosaccharides can cross-feed to support growth of specific commensal gut bacteria.
2. Infant gut commensal bacteria co-evolved with HMO structures, and the capacity to utilize different HMOs varies among commensal bacteria (18, 19). Therefore, increasing the number and structural diversity of HMOs is expected to support a wider array of beneficial bacteria, which consequently beneficially impacts the gut metabolic profile. This concept is supported by data from an infant fecal fermentation study that systematically compared the effects of one, two and six HMOs on the infant microbiota (20).
3. HMOs have a variety of anti-pathogenic effects, including inhibiting growth and colonization of pathogens that cannot utilize HMOs as growth substrates (competitive exclusion), functioning as decoy receptors for pathogenic viruses and bacteria, and modulating the neonatal immune system through direct interactions with epithelial and immune cells (21, 22). Due to the structural specificity of HMO binding interactions with receptors on human cells and with pathogens, supplementation with a greater number of HMOs is expected to support defense against a broader array of viruses and bacteria.
4. Data from observational human studies and experimental animal models indicate that fucosylated and sialylated HMOs could play important roles in brain development and cognitive function and that these different classes of HMOs function through distinct mechanisms (23). Preclinical studies show that sialic acid derived from sialylated HMOs can cross the blood-brain barrier, whereas fucose cannot, and fucosylated HMOs appear to function through the microbiome-gut-brain axis.

### 2.1 Degradation of HMOs from different structural classes releases different monosaccharides

There are five monosaccharide building blocks that comprise HMOs: glucose, galactose, fucose, GlcNAc and sialic acid. HMOs contain lactose at the reducing end and are covalently bound to one or more monosaccharides or disaccharides. Lactose can be fucosylated or sialylated through different linkages to generate the smallest trisaccharide HMOs: 2′-FL, 3-FL, 3′-SL and 6′-SL. Lactose can also be extended by addition of the disaccharides lacto-N-biose [galactose bound to GlcNAc through a β1-3 linkage, as found in lacto-N-tetraose (LNT)] and/or N-acetyllactosamine (galactose bound to GlcNAc through a β1-4 linkage, as found in LNnT). Lactose can be elongated by one or more of these disaccharides in either a linear or branching pattern and can be further modified by addition of one or more fucose and/or sialic acid residues through various glycosidic linkages. These possibilities for HMO synthesis generate a pool of diverse structures that vary in size, composition and conformation (see examples in Table 1). Depending on their monosaccharide composition, HMOs are generally classified as belonging to one of three major structural classes: fucosylated, neutral core or sialylated, although fucosylated acidic HMOs (i.e., HMOs that contain both fucose and sialic acid molecules) also exist and may be considered a hybrid of the fucosylated and sialylated structural classes. Depending on structural class, different molecules can be released following degradation of HMOs by the gut microbiota, including fucose, GlcNAc and sialic acid. These three molecules have unique structures and biological functions and can be further utilized by intestinal bacteria to produce specific secondary metabolites, affect intestinal function, or be absorbed and incorporated in distal organs such as the brain.

**Table 1:** Examples of the structural diversity and complexity of HMOs.

	Fucosylated HMOs		Neutral core HMOs		Sialylated HMOs	
Trisaccharides	2′-FL	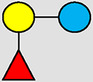	LNT2^a^	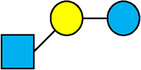	3′-SL	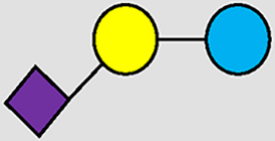
	3-FL	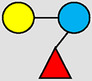			6′-SL	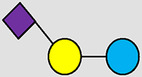
Tetrasaccharides	DFL (LDFT)	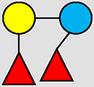	LNT	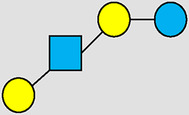	3′-S-3-FL^b^	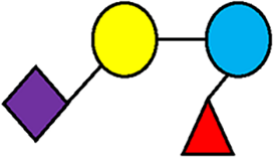
			LNnT	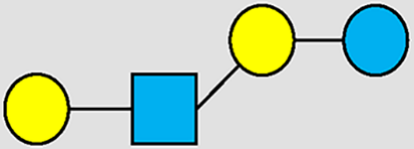		
Pentasaccharides	LNFP-I	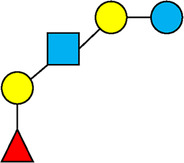	LNH	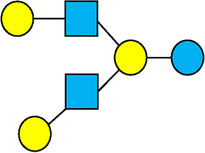	LSTa	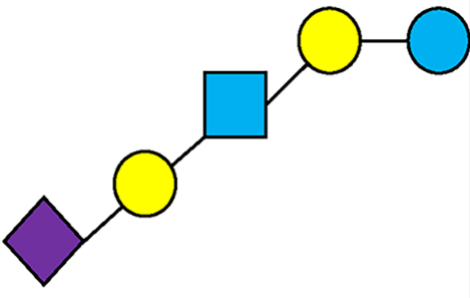
	LNFP-II	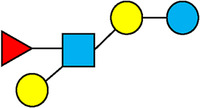	LNnH	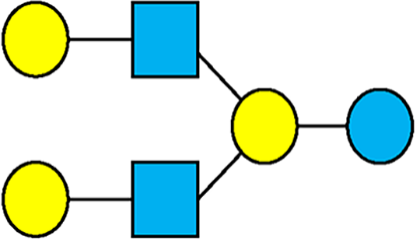	LSTb	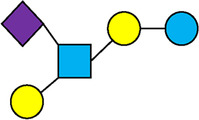
	LNFP-III	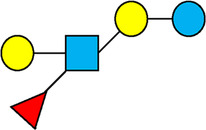			LSTc	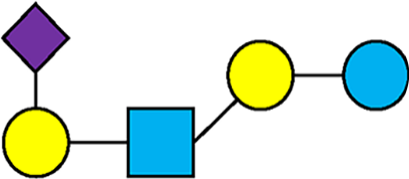
Hexasaccharides	LNDFH-I	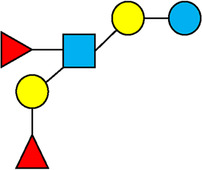	*para*-LNH	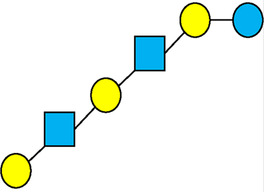	DSLNT	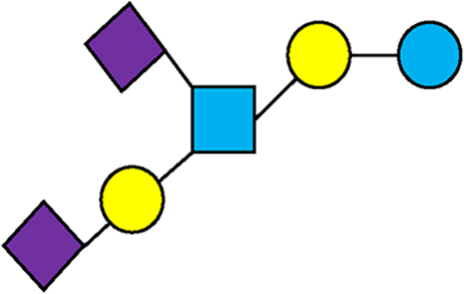
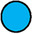 , Glucose; 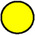 , Galactose; 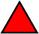 , Fucose; 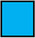 , N-Acetylglucosamine; 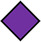 , N-Acetylneuraminic acid.						

The table shows examples of HMO structures organized by monosaccharide number and composition, including the 10 most abundant HMOs in breastmilk. Manufactured HMOs that are currently commercially available are indicated by gray background. The table shows only a subset of the more than 160 HMO structures that have been elucidated and focuses on the smaller and simpler structures. It should be noted that HMOs containing up to 14 monosaccharide units have been elucidated (24).

HMO Abbreviations: 2′-FL, 2′-fucosyllactose; 3-FL, 3-fucosyllactose; 3′-S-3-FL, 3′-sialyl-3-fucosyllactose; 3′-SL, 3′-sialyllactose; 6′-SL, 6′-sialyllactose; DFL, Difucosyllactose; DSLNT, Disialyllacto-N-tetraose; LDFT, Lactodifucotetraose; LNDFH-I, Lacto-N-difuco-hexaose I; LNFP-(I-III), Lacto-N-fucopentaose (I-III); LNH, Lacto-N-hexaose; LNnH, Lacto-N-neohexaose; LNnT, Lacto-N-neotetraose; LNT, Lacto-N-tetraose; LST(a-c), LS-Tetrasaccharide (a-c); *para*-LNH, *para*-Lacto-N-hexaose.

^a^ LNT2 is a precursor for HMO synthesis and an intermediate in HMO degradation, particularly for LNT and LNnT.

^b^ 3′-S-3-FL is an example of an HMO that is both fucosylated and sialylated.

Fucosylated HMOs account for the greatest proportion of the total HMO pool, typically at 60%-70%, with some variation according to geographic location and genetic factors (25). The majority of the global population, ranging from 65%–98% in different geographic locations (26), have a functional 2-α-L-fucosyltransferase 2 (FUT-2) gene, produce high levels of α1,2-fucosylated HMOs such as 2′-FL and LNFP-I, and are referred to as secretors. In contrast, non-secretors have polymorphisms that render FUT-2 non-functional and produce little to no α1,2-fucosylated HMOs, although they can produce α1,3-fucosylated HMOs such as 3-FL and LNFP-III. Given the differences in the types and total concentrations of fucosylated HMOs in the breastmilk of secretors and non-secretors, several studies have investigated whether mothers’ secretor status affects infants’ gut microbiota composition. Two studies that sampled during the early lactation period (6 to 120 or 180 days post-partum) in North American and Chinese breastfed infants found that bifidobacteria were established earlier and at higher levels in infants of secretor mothers (27, 28). However, studies of Indonesian, Finnish and Danish breastfed infants sampled at 60–65 days, 3 months and 5 months, respectively, reported that maternal secretor status was not associated with infants’ microbiota composition and that there were no significant differences in the relative abundance of bifidobacteria (29–31). One exception was the caesarean-born subgroup of the Finnish cohort, where the infants of non-secretors had fewer bifidobacteria and more enterococci (30). Thus, a consistent effect of secretor status on infant microbiota composition cannot be concluded and is likely challenged by heterogeneity in the study cohorts with regards to sample population, birth mode, lactation period and the extent to which infants are exclusively breastfed.

Fucose that is liberated by degradation of fucosylated HMOs can be further metabolized by infant gut-associated bacteria such as *Bifidobacterium longum* subsp. *infantis* and *Bifidobacterium longum* subsp. *suis* to produce 1,2-propanediol (32), which can cross-feed to other gut commensal bacteria such as *Anaerobutyricum hallii* (formerly *Eubacterium hallii*) to produce propionic acid (33). Short chain fatty acids such as propionic acid are important cellular energy sources and cell signaling molecules. Fucose may also support growth of diverse gut bacteria, as several species of *Bacteroides* and specific strains of *Lacticaseibacillus rhamnosus* have been shown to grow on fucose as a sole carbon source (34). In addition to supporting trophic interactions of the microbiota, fucose has also been shown to suppress virulence genes in pathogens such as *E. coli*, and fucosylated glycans have been shown to protect against intestinal and systemic inflammation in animal models (35). By providing a source of free fucose in the intestine, fucosylated HMOs appear to play multiple roles in shaping the gut microbiota and maintaining intestinal homeostasis.

Like fucose, GlcNAc supports trophic interactions in the complex ecosystem of the gut microbiota. Liberating GlcNAc from neutral core HMOs such as LNnT and LNT requires degrading both the linkage of the terminal galactose to GlcNAc and the linkage of GlcNAc to the lactose core. Several species of infant gut-associated bifidobacteria including *B. infantis* and *B. bifidum* possess the necessary enzymes to degrade these linkages (36). The enzymatic capability to degrade lacto-*N*-triose II (LNT2, a degradation intermediate of LNnT and LNT) to liberate GlcNAc and lactose has been demonstrated for *B. breve* UCC2003 (37). Subsequently, free GlcNAc can potentially be utilized by other gut bacteria, as it has been demonstrated that specific strains of *Lactobacillus* species such as *L. plantarum* and *L. paracasei* can grow on GlcNAc as a sole carbon source (38). In addition, an animal study showed that oral supplementation with GlcNAc enhanced intestinal barrier function and protected against chemically-induced colitis (39). A pilot clinical study that tested oral administration of GlcNAc in children with treatment-resistant inflammatory bowel disease showed promising results in improvement of symptoms (40). These results suggest that GlcNAc itself may influence barrier function and inflammation in the intestine.

Sialylated HMOs are typically present at 10%–20% of the total HMO pool, with geographic variation (25). Degradation of sialylated HMOs, such as 3′-SL and 6′-SL, by intestinal bacteria releases sialic acid. In contrast to fucose and GlcNAc, which can be utilized for growth by several *Bacteroides* and *Lactobacillus* species as described above, the ability to utilize sialic acid appears to be uncommon among typical infant gut-associated bacteria. In a screen of 12 *Bifidobacterium* strains and 12 *Lactobacillus* strains, only one strain, *L. plantarum* LP-66, was able to grow on sialic acid as a sole carbon source, whereas half of the strains were able to grow on GlcNAc (38). This apparent limited capacity to utilize sialic acid may mean that it is more available to be absorbed into circulation and incorporated into distal organs such as the brain.

### 2.2 Combining structurally diverse HMOs should support a wider array of beneficial bacteria

Typically, the most abundant bacterial species found in the breastfed infant gut microbiota are *Bifidobacterium* species, including *B. longum* subsp*. infantis*, *B. bifidum*, *B. longum* subsp*. longum* and *B. breve* (41, 42). While *B. longum* subsp*. infantis* and *B. bifidum* possess many glycoside hydrolase enzymes and can degrade a wide range of structurally distinct HMOs, other species commonly associated with the breastfed infant gut microbiota, such as *B. longum* subsp. *longum* and *B. breve,* generally can degrade only neutral core HMOs, especially LNT [reviewed in (43); summarized in Table 2].

**Table 2:** General trends for utilization of HMOs by infant gut-associated *Bifidobacterium* species.

*Bifidobacterium* species	2′-FL	3-FL	DFL	LNT	LNnT	3′-SL	6′-SL	References
*B. longum* subsp. *infantis*	**+**	**+**	**+**	**+**	**+**	**+**	**+**	(32, 34, 38, 44–46)
*B. bifidum*	**+**	**+**	**+**	**+**	**+**	ND	**+**	(32, 38, 44)
*B. longum* subsp. *longum*	**−**	**−**	ND	**+**	±	**−**	**−**	(45)
*B. breve*	**−**	**−**	ND	**+**	**+**	**−**	**−**	(47)

+, growth on HMO as a sole carbon source.

**−**, no growth on HMO as a sole carbon source.

±, growth on HMO is highly variable among strains of this species.

ND, no data.

In a screen of 21 *B. longum* subsp*. infantis* strains and 13 *B. bifidum* strains tested for their ability to grow on each of five individual HMOs provided as the sole carbon source, all the *B. longum* subsp*. infantis* strains and the majority of *B. bifidum* strains grew on 2′-FL, 3-FL, LNT, LNnT and 6′-SL (44). In contrast, of 17 *B. longum* subsp. *longum* strains tested for their ability to grow on each of six individual HMOs, only one strain grew on 2′-FL and 3-FL, none of the strains grew on 3′-SL or 6′-SL, and the capacity to grow on LNnT was highly variable (45). However, all but one of the 17 *B. longum* subsp. *longum* strains exhibited high growth on LNT (45). Similar to *B. longum* subsp. *longum*, a screen of 24 *B. breve* strains showed that the ability to grow on 2′-FL or 3-FL was rare (high growth on 2′-FL for only two of 24 strains tested), and none of the strains grew on 3′-SL or 6′-SL (47). However, all 24 *B. breve* strains exhibited high growth on LNT and LNnT. Despite this relatively limited capacity to degrade HMOs, *B. breve* is frequently identified in infant stool samples, which is attributed to its ability to function as a scavenger species that can cross-feed on HMO-derived monosaccharides (43). The *in vitro* data suggest that inclusion of neutral core HMOs is likely to support growth of infant gut-associated *B. longum* subsp. *longum* and *B. breve* species to a greater extent than what would be expected for fucosylated or sialylated HMOs.

In addition to *Bifidobacterium* species, other infant gut commensal bacteria are capable of utilising HMOs, including several *Bacteroides* species and *Akkermansia muciniphila* (46, 48–50). Although these species are typically less abundant in the breastfed-infant gut microbiota compared to *B. infantis* and *B. bifidum*, they may nevertheless play important and specialized roles, for example in metabolite production, immune modulation, and mucin degradation (51). Since *Bacteroides* are generally equipped with diverse enzymes for degrading complex plant polysaccharides (52), they may play a key role during the transition to solid foods. Although the capability to utilize HMOs is quite limited among *Lactobacillus* species, some lactobacilli can cross-feed on HMO-derived monosaccharides, as described in the previous examples. Thus, as the number and structural diversity of HMOs increases, it is expected that HMOs and their degradation products will support growth of a wider array of beneficial gut bacteria.

Results of a recent infant fecal fermentation study support this hypothesis. The effects of 2′-FL alone, 2′-FL+LNnT, and a 6 HMO mix (containing 2′-FL, DFL, LNT, LNnT, 3′-SL and 6′-SL) on the infant microbiota were compared in a long-term *in vitro* fermentation study that featured a 2-week stabilization period, 2-week baseline period and 3-week treatment period (20). Although all HMO treatments promoted increases in *Bifidobacteriacaea,* the 6 HMO mix promoted the highest abundance and greatest diversity of *Bifidobacteriacaea*. In addition, the 6 HMO mix was the only treatment that promoted increases in *Bacteroidaceae* at the end of the treatment period, as well as increases in beneficial butyrate-producing bacteria such as *Faecalibacterium prausnitzii*. Furthermore, filtered supernatant from only the 6 HMO mix significantly enhanced intestinal barrier function *in vitro*, indicating that metabolites produced by a microbiota supplemented with a complex mixture of HMOs have more substantial positive effects on intestinal physiology.

These preclinical data on the effects of HMOs on the infant gut microbiota are substantiated by results from several clinical intervention studies, which reported that supplementing infant formulae with mixtures of 2 or 5 HMOs brings the gut microbiota composition closer to that of breastfed infants (summarized in Table 3). In one study, infants receiving infant formula supplemented with 2′-FL, DFL, LNT, 3′-SL and 6′-SL at total doses of either 1.5 or 2.5 g/L had higher relative abundances of total *Bifidobacterium* and *B. longum* subsp. *infantis,* which were significantly higher than the control group and similar to or approaching the breastfed reference group (59). Significant differences between the HMO test groups and the control formula group were also observed for several other bacterial taxa including Clostridia, *Lactobacillus*, Peptostreptococcaceae, and *Streptococcus*. For example, the test group receiving the higher HMO dose had a significantly higher relative abundance of *Lactobacillus,* approaching that of the breastfed group (59). In another clinical study, infants receiving infant formula supplemented with 2′-FL, 3-FL, LNT, 3′-SL and 6′-SL at a total dose of 5.75 g/L had higher relative abundances of *Bifidobacterium*, particularly *Bifidobacterium* species capable of producing aromatic lactic acids, and lower relative abundances of *Escherichia* and *Enterococcus*, compared to the control group (57). Importantly, there were notable differences in the abundances of *Bifidobacterium* (sub)-species observed in a clinical trial that evaluated the effects of infant formula supplemented with 7.2 g/L bovine milk-derived oligosaccharides (BMOS), consisting of majority GOS and 0.06 g/L sialylated oligosaccharides (60). The group receiving BMOS had higher relative abundances of *B. dentium*, *B. longum* subsp. *longum*, and *B. breve*, whereas the breastfed reference group had higher relative abundances of the efficient HMO-utilizing species *B. longum* subsp. *infantis* and *B. bifidum*, indicating that GOS does not modulate the infant gut microbiota with the same specificity as HMOs.

**Table 3:** Randomized controlled clinical trials that evaluated combinations of manufactured HMOs in infants and reported physiological outcomes related to gut microbiota and immunity.

References	Study Population and Duration	Total HMO Dose	Key Results in Test Formula Group(s) Compared to Control Formula Group
2′-FL and LNnT			
(53, 54)	Healthy term infants Formula groups: *n* = 87–88/group Breastfed reference group: *n* = 35 6-month intervention with follow-up until 12 months of age	1.5 g/L	**Gut Microbiota** • SS ↓ phylogenetic diversity at 3 months, approaching breastfed group • SS ↑ relative abundance of *Bifidobacterium* and SS ↓ *Escherichia*, comparable to breastfed group • SS ↑ number of infants with *Bifidobacteriaceae* at high abundance (92%) (also predominant in breastfed group) **Infection/Immune-related** • SS ↓ bronchitis through 4, 6, and 12 months • SS ↓ lower respiratory tract infection through 12 months • SS ↓ use of antipyretics through 4 months • SS ↓ use of antibiotics through 6 and 12 months
(55, 56)	Term infants with cow's milk protein allergy Formula groups: *n* = 97/group 4-month intervention with voluntary continuation up to 12 months of age	1.5 g/L	**Gut Microbiota** In early enrollment (≤ 90 days) cohort: • SS ↓ phylogenetic diversity after 3 months of treatment • SS ↑ relative abundance of *Bifidobacterium* after 1 and 3 months of treatment • SS ↓ proportion of infants with ‘FCT3′ microbiota composition, indicating delayed maturation of the microbiota toward an adult-like composition **Infection/Immune-related** • SS ↓ in frequency of upper respiratory tract infections and incidence of ear infections at 12 months of age • Reduction in relative risk of lower respiratory tract infections and gastrointestinal infections by 30–40% at 12 months of age, but NSD due to sample size limitations • NSD in antibiotic use between groups • SS ↓ antipyretic use between 4-month follow-up and 12 months of age
5 HMO mixes (2′-FL, LNT, 3′-SL, 6′-SL and either 3-FL^a^ or DFL^b^)			
(57, 58)^a^	Healthy term infants Formula groups: *n* = 112–113/group Breastfed reference group: *n* = 116 4-month intervention	5.75 g/L	**Gut Microbiota** • SS ↑ relative abundance of *Bifidobacterium* at Weeks 1, 2, 4 and 16 • SS ↑ in *Bifidobacterium* with aromatic lactate dehydrogenase at Weeks 1 and 16 • SS ↓ relative abundance of *Escherichia* and *Enterococcus* • SS ↓ relative abundance of opportunistic pathogens at Weeks 1, 2 and 4
(59)^b^	Healthy term infants Formula groups: *n* = 230–233/group Breastfed reference group: *n* = 96 15-month intervention	1.5 g/L; 2.5 g/L	**Gut Microbiota** • Alpha diversity indexes (richness and Shannon index at genus level) were lower at 6 months, approaching breastfed group • −45% ↑ *Bifidobacterium* abundance at 6 months, approaching breastfed group • SS ↑ relative abundance of *Bifidobacterium longum* subsp*. infantis* at 3 and 6 months, approaching breastfed group • SS ↑ relative abundance of *Lactobacillus* (test group with 2.5 g/L HMOs), approaching breastfed group • SS ↓ abundance of toxigenic *Clostridioides difficile* at 3 and 6 months **Gut Metabolites** • SS ↓ fecal pH at 3 and 6 months • Overall organic acid profile approaching breastfed group **Infection/Immune-related** • SS ↑ secretory immunoglobulin A at 3 months • SS ↓ faecal calprotection at 6 months (test group with 1.5 g/L HMOs) • SS ↓ faecal alpha-1-antitrypsin at 3 months

NSD, no significant difference; SS, statistically significant.

^a^ 5 HMO mix containing 2'-FL, 3-FL, LNT, 3'-SL, 6'-SL.

^b^ 5 HMO mix containing 2'-FL, DFL, LNT, 3'-SL, 6'-SL.

### 2.3 Increasing the number of HMOs may support defense against a broader range of pathogens

Systematic reviews have concluded that exclusive breastfeeding reduces the risk of gastrointestinal and respiratory infections (8, 9). Among the many bioactive components in breastmilk that may protect against infections, HMOs are important contributors. One mechanism by which HMOs support defense against infections is modulation of the neonatal immune system. HMOs share structural similarities with blood group glycans and can bind to several classes of lectins, including C-type lectins, galectins and siglecs, which are found on the cell surface of different human cell types, especially immune cells (22). Due to the structural specificity of HMO-lectin interactions, different HMOs would be expected to have different immunomodulatory effects. For example, C-type lectins like DC-SIGN, which is expressed on the surface of dendritic cells, have high specificity for α-fucosylated glycans and have been shown to bind 2′-FL and 3-FL, but not LNT (61). Galectins, which are expressed by intestinal epithelial cells, lymphocytes and antigen-presenting cells, exhibit specificity for glycans containing N-acetyllactosamine and basic disaccharide units such as Galβ1-3/4GlcNAc (22, 62), which are found in neutral core HMOs such as LNT and LNnT. Siglecs are expressed on multiple immune cell types and exhibit specific binding affinity to sialyllactoses (22, 61).

Although relatively few studies to-date have compared the immunomodulatory potential of several different HMOs, a recent study that evaluated six different HMOs on several *in vitro* models of human intestinal epithelial and immune cells reported some differential effects of HMOs. Whereas sialylated HMOs (3′-SL, 6′-SL) and 3-FL stimulated cytokine release from LPS-activated dendritic cells and M1 macrophages, neutral core HMOs (LNT, LNT2) and fucosylated HMOs (2′-FL, 3-FL) had the most pronounced effects on enhancing intestinal barrier integrity of epithelial cells (63). Animal model data support that combinations of HMOs affect immune cell populations. Compared to newborn piglets fed a control sow milk replacer formula, piglets who received formula supplemented with 4 g/L HMOs (including 2′-FL, LNnT, 6′-SL, 3′-SL and free sialic acid) had significantly more circulating natural killer cells and effector memory T cells in mesenteric lymph nodes, both under noninfected and rotavirus-infected conditions (64). HMO-fed piglets also exhibited reduced duration of rotavirus-induced diarrhea (65). Piglets that received 4 g/L of a combination of GOS and FOS displayed intermediate levels of these immune cell populations, suggesting that HMOs may have more specific and pronounced immunomodulatory effects (64). In another study, newborn piglets received control formula or formula supplemented with either 6.5 g/L BMOS (majority GOS plus 3′-SL and 6′-SL, as described previously), 1.5 g/L of HMOs (2′-FL and LNnT), or the combination of BMOS and HMOs. While piglets that received only BMOS or only HMOs displayed the same percentage of PBMC T-helper cells as the control formula group, piglets that received the combination of BMOS and HMOs had a significantly lower PBMC T-helper cell population (66). These findings are consistent with results from studies of both human infants and piglets, where groups that received mother's milk had significantly lower PBMC T-helper cell populations compared to formula-fed groups (67, 68). Further research is warranted to gain a better understanding of the immunomodulatory potential of HMOs, both as individual structures and in combinations.

In addition to their ability to modulate the immune system, HMOs can function as decoy receptors, binding infectious pathogens and their toxins and thereby blocking pathogen adhesion and infection of host cells (21, 69). Due to structural constraints of receptor binding domains, these interactions are very specific between a particular pathogen and one or a limited number of (often structurally related) HMOs. In mapping the literature of known HMO-virus interactions, a broad trend emerges for HMOs of different structural classes binding to different virus families/genera (see Table 4), although it should be noted that exceptions to this general trend do exist. For example, fucosylated HMOs such as 2′-FL, but not neutral core HMOs (LNT and LNnT), have been shown to bind to several different norovirus strains (70, 74), whereas LNT and LNnT have been shown to bind to several different rotavirus strains (77, 79, 80). For the sialylated HMOs, the weight of evidence for virus binding is for respiratory viruses, including influenza viruses, RSV and mumps virus (81, 82, 84). Since the nasopharyngeal tract is bathed in milk during breastfeeding, it has been postulated that low levels of HMOs may be present in the nasopharynx (88), and this hypothesis is supported by the observation of a low abundance of *Bifidobacterium* in the nasal microbiome of neonates (89). Thus, HMOs that are retained in the nasopharynx or that travel to the respiratory tract via the circulation upon absorption from the intestine may be able to support direct inhibition of respiratory pathogens in the respiratory tract.

**Table 4:** Interactions between viruses and HMOs demonstrated in binding assays and structural analyses.

Virus	Reference	Strain/Virus-Like particle	2'-FL	3-FL	DFL	LNT	LNnT	6'-SL	3'-SL	Assay/Notes
Noroviruses	(70)	VA387 (GII.4)	✓	X	X	X	X			Binding assay. While 2'-FL and DFL bound the Norwalk capsid with moderate affinity, LNFP I and LNDFH I bound with high affinity.
		Norwalk capsid (GI.1)	✓	X	✓	X	X			
	(71)	GII.10	✓	✓						Inhibition of binding to surrogate HBGA samples
	(72)	GI.1	✓							Binding assay
		GII.17	✓							
	(73)	GII.4							✓	Binding assay. LNnFP I and LNnFP V also bound to GII-17.
	(74)	GII.17	✓	✓		X	X			
Rotaviruses	(75)	sialidase-sensitive porcine strain OSU	X				X	✓	✓	Inhibition of rotavirus infectivity and binding to MA104 cells
		sialidase-insensitive human strain Wa G1P[8]	X				X	X	X	
	(76)	VP8* domain of RV3 [G3P[6]]				✓				Rotavirus VP8* domain binding to human milk glycan microarray.
	(77)	VP8* of neonate-specific G10P[11]				✓	✓			Binding assay
	(78)	human strain G1P[8]	✓					✓	✓	Inhibition of rotavirus infectivity of MA104 cells
		human strain G2P[4]	✓					✓	✓	
	(79)	VP8* domain of P[19]				✓				Binding assay
		VP8* domain of P[6]				✓				
		VP8* domain of P[4]				X				
		VP8* domain of P[8]				X				
	(80)	VP8* domain of G10P[11]				✓	✓			LNT, LNnT and pooled HMOs enhance neonatal rotavirus G10P[11] infectivity of MA104 cells. Pooled HMOs do not enhance infectivity of human rotavirus strains G1P[8](strain Wa) or G2P.
		Rotavac® vaccine: G9P[11]	✓			✓		X		Ability to enhance infectivity of Rotavac® P[11] vaccine in MA104 cells
Influenza viruses	(81)	Influenza A H1N1 strain	X				✓	✓	X	Reduction of viral load in 16HBE epithelial cells
	(82)	Avian influenza viruses 6 subtypes, 13 strains						8/13	13/13	Hemaaglutination inhibition assay
Respiratory syncytial virus	(81)	RSV A strain	✓				X	X	✓	Reduction of viral load in 16HBE epithelial cells
Mumps virus	(83)	MuV-HN head domain							✓	Crystal structure of mumps virus attachment protein hemagglutinin-neuradminidase (MuV-HN) in complex with 3'-SL.
	(84)	MuV-HN proteins					X	X	✓	Large scale glycan array screen with MuV-HN attachment proteins. 3'-SL inhibited MuV entry into Vero cells.
	(85)	MuV-HN protein							✓	Structural analyses show that 3'-SL forms a stable complex with the binding cavity of the HN protein of the mumps virus through specific attractive and hydrogen bond interactions.
SARS-CoV-2	(86)	Whole virus	X				✓	✓		Inhibition of infection and cytotoxicity of whole SARS-CoV-2 in Vero E6 cells
		Spike RBD protein	X				✓	✓		Inhibition of RBD binding to HepG2 cells. 6'-SL was more effective than LNnT at blocking binding.
	(87)	Spike RBD protein	✓	X				X	X	Inhibition of RBD protein binding to Vero E6 cells (high ACE2 expressing)
			✓	✓				X	X	Inhibition of RBD protein binding to Calu-3 cells (low ACE2 expressing)

The shading of the HMOs indicate structural class as follows: light pink, fucosylated HMOs; blue, neutral core HMOs; purple, sialylated HMOs. The shading of the cells indicates the following: dark green, HMO binds and/or inhibits infectivity of virus (see Assay/Notes for details); light green, HMO has moderate affinity interaction with virus (in contrast to high affinity interaction demonstrated for other HMOs); red, no HMO-virus interaction; gray, interaction not tested. References (70–87).

Beyond the broad pattern of different structural classes of HMOs binding to different virus families/genera, there are important details regarding the specificity of HMO-virus interactions. For example, 6′-SL, but not 3′-SL, could reduce viral load of an Influenza A H1N1 strain in 16HBE epithelial cells, whereas 3′-SL, but not 6′-SL, could reduce viral load of an RSV A strain in the same cell type (81). These data suggest that the interactions are not generic to all sialylated HMOs, but rather very specific to the particular HMO structures and the virus strains, constituting a primary example for the common concept of glycan evolution in light of a host-pathogen arms race (90, 91). Similarly, when two different norovirus strains were tested against a panel of fucosylated and neutral core HMOs, the VA387 (GII.4) strain bound with high affinity only to 2′-FL and LNFP-III, whereas the Norwalk capsid (GI.1) bound with high affinity to LNFP-I and LNDFH-I (70). Therefore, to support defense against a broader range of viral pathogens, supplementation with multiple HMOs with diverse structures is likely to be beneficial.

In addition to these specific HMO-virus interactions, HMOs also have specific and differential interactions with pathogenic bacteria, bacterial toxins and parasites [reviewed in (5)]. For example, in a screen of 16 individual HMOs, only DFL and one other HMO could reduce growth of *Streptococcus agalactiae* (Group B Strep, a significant pathogen for pregnant women and newborns) by more than 50% (92). Despite their structural similarity to DFL, 2′-FL and 3-FL reduced growth by only 8% and 15%, respectively. This study also showed that a heterogeneous extract of HMOs could reduce growth of *S. agalactiae* by 82%, underscoring the potential for greater biological effects with blends of many HMOs compared to individual HMOs. In another notable example, only five of 20 individual HMOs, including 2′-FL, LNT and LNFP I-III, could bind to both toxin A (TcdA) and toxin B (TcdB) of *Clostridioides difficile* (93), and both 2′-FL and LNnT could inhibit growth of *C. difficile* in *in vitro* fecal fermentation models (94, 95). Clinical data from HMO intervention studies corroborate these preclinical findings, as infants who received infant formula supplemented with either 2′-FL or a mix of 5 HMOs (including 2′-FL, DFL, LNT, 3′-SL and 6′-SL) had relative abundances of *C. difficile* that were significantly lower than the control formula groups, and comparable with the breastfed reference groups (59, 96). Current knowledge on HMO-pathogen interactions may be biased by which HMOs have been most readily available and therefore most studied. Although some studies of HMO-pathogen interactions focused on a limited number of HMOs due to known structural interactions (e.g., influenza viruses use sialic acid receptors to invade host cells), many studies likely tested a small number of HMO structures due to limited availability. Now that HMO manufacturers can produce many HMOs at high purity and in reasonable quantities, systematic testing of larger panels of HMOs could reveal more insights into the differential roles of HMOs in pathogen inhibition.

The preclinical data indicating that HMOs have immunomodulatory and anti-pathogenic effects are supported by some clinical data showing that combinations of HMOs may enhance defense against infections (summarized in Table 3). Two clinical trials that evaluated the combination of 2′-FL and LNnT reported statistically significant reductions in respiratory tract infections and antipyretic use in the groups that received HMOs as compared with controls (53, 55). HMO supplementation has also been reported to modulate biomarkers of immune function. Fecal samples from infants who received infant formula supplemented with a 5-HMO mix exhibited significantly higher levels of secretory immunoglobulin A and significantly lower levels of calprotectin compared with the control group (59). Further clinical research is needed to substantiate these results and to better understand which combinations of HMOs can exert the most beneficial effects on immune system development and defense against infections. However, attributing reduced infection incidence to an infant formula ingredient can be challenging, as it requires sufficient infection incidence in the study population and an adequately powered sample size in order to show a statistically significant difference between control and test groups.

### 2.4 Fucosylated and sialylated HMOs may contribute to brain development and cognitive function through distinct mechanisms

The effect of breastfeeding on cognitive development has been extensively studied, and several systematic reviews have concluded that breastfeeding is positively associated with improved performance on intelligence tests, even when correcting for confounding factors such as maternal IQ and home environment (10, 11). Breastmilk contains multiple bioactives that may contribute to neurodevelopment, including fucosylated and sialylated HMOs (23). Sialic acid is an essential building block of brain gangliosides, and in humans, the highest concentration of sialic acid is found in the brain (97). Free sialic acid and sialic acid derived from dietary sialyllactose can cross the blood-brain barrier and be incorporated into brain tissue, as demonstrated by isotope-labeled studies in rodent pups (98, 99). Animal studies suggest that sialic acid derived from sialyllactoses is more slowly released and more readily incorporated in tissues compared to free sialic acid, which is quickly excreted into urine (100, 101). In contrast to sialylated HMOs, animal studies with isotope-labelled 2′-FL indicate that fucosylated HMOs may influence cognitive development through indirect mechanisms involving the gut microbiota and the gut-brain axis. Oral administration of ^13^C-labelled 2′-FL resulted in ^13^C-enrichment in plasma, brain and other organs in wild-type mice, but not in germ-free mice where ^13^C remained in intestinal and fecal samples, suggesting that microbial metabolism of 2′-FL is necessary for absorption and incorporation into organs (102). A follow-up study with ^13^C-labelled fucose indicated that, although fucose was rapidly absorbed into plasma after oral dosing, ^13^C-enrichment in the brain did not occur until after more than 3 h, coinciding with arrival of the ^13^C-fucose bolus in the colon (103). Furthermore, neither ^13^C-2′-FL nor ^13^C-fucose administered intravenously resulted in ^13^C-enrichment in the brain, indicating than circulating 2′-FL and fucose do not cross the blood-brain barrier (102, 103).

Observational studies in breastfed infants have indicated positive associations between specific HMOs and various aspects of cognitive development. Several positive associations have been reported for sialylated HMOs and cognitive outcomes. In particular, concentrations of 3′-SL have been positively associated with early language learning scores (104), and concentrations of 6′-SL at 1 month of age have been positively associated with cognitive and motor scale scores at 18 months (105). Observational studies have also found positive associations between 2′-FL concentrations and improved cognitive outcomes, including positive motor scores at 6 months of age (105), higher cognitive development scores at 24 months (106), and better executive functioning at three years of age (107). To date, most observational studies that have evaluated cognitive outcomes have focused on associations with individual HMOs. However, a few studies have reported positive associations of total fucosylated HMOs or total sialylated HMOs, for example with language development at 18 months and executive functioning at three years old (107, 108). Although these data are generally supportive, results of observational studies should be interpreted with caution, since cognitive development is a complex, multifaceted process that is influenced by many factors, including genetics and environment. Limitations of these studies include imprecise data on breastmilk/HMO intake and assessment of cognitive outcomes based on parent-reported questionnaires. Thus, experimental animal studies, where genetics, environment, HMO dosing, and assessment of cognitive function can be highly controlled, are an important tool for investigating the effects of individual and multiple HMOs on cognitive development. These preclinical studies were recently reviewed in depth (23), but key results will be highlighted here.

Piglets are an established model for studying the influence of pediatric nutrition on neurodevelopment, since piglets and human infants have similar nutritional requirements and brain development patterns (109). Consistent with results of HMO intervention trials in human infants, supplementation of milk replacer formula with 2′-FL, 3′-SL or 6′-SL individually or with bovine-derived whey enriched with sialyllactose was well-tolerated and supported normal growth of piglets (17, 110–112). Studies of piglets supplemented with sialylated HMOs have yielded mixed results with regards to cognitive outcomes. A few studies have found positive effects of sialylated HMOs on memory and learning. For example, among piglets delivered preterm, a higher proportion of those who were supplemented with sialyllactoses (3′-SL + 6′-SL) reached T-maze learning criteria compared to controls (113). In addition, genes related to sialic acid metabolism, myelination, and ganglioside biosynthesis were upregulated in the hippocampus of piglets who received sialyllactoses. In another study, minipigs supplemented with sialyllactoses in milk formula demonstrated improved reference memory during reversal learning in the period corresponding to adolescence, suggesting that sialyllactoses may promote cognitive flexibility (114). However, sialyllactose supplementation did not affect the performance of minipigs on several other cognitive tests, including open field, novel object recognition and runway tasks. Two additional studies also reported that there were no differences in performance on novel object recognition tests between control and test groups of piglets supplemented with either 3′-SL, 6′-SL or sialyllactose (115, 116). These discrepancies might be explained by the fact that the different cognitive tests used to assess cognitive development require different neural pathways (e.g., spatial awareness versus exploration of novelty). It is possible that sialyllactose supplementation supports development of specific neural pathways and therefore enhances performance only on some types of cognitive tasks.

In addition to the studies in piglets, the effects of 3′-SL and 6′-SL on cognition have also been evaluated in rodents. Rat pups who received 6′-SL during the lactation period demonstrated significantly improved long-term potentiation (LTP; the persistent strengthening of synapses between neurons in the brain that is involved in learning and memory) at one year of age, indicating that early life exposure to 6′-SL had long-term benefits on learning and memory (117). As a complement to the studies on sialyllactose supplementation, several mouse knockout model studies have shown that deficiencies in sialylated oligosaccharide synthesis in dams result in reduced cognitive abilities in fostered offspring. Mouse pups deprived of 3′-SL, 6′-SL or both during lactation exhibited reduced cognitive functions in adulthood, such as decreased spatial and working memory (118–120).

Similar to the findings of 3′-SL and 6′-SL supplementation in piglets, dietary administration of 1 g/L 2′-FL, either alone or in a synbiotic combination with *B. longum* subsp. *infantis* Bi-26, did not affect novel object recognition memory after 48 h. However, the 2′-FL group had a higher number of object visits, and both the 2′-FL and 2′-FL + Bi-26 groups exhibited a significantly larger relative volume of the pons region of the brain, indicating effects of 2′-FL on both brain structure and behavior (121). Interestingly, in a similar study that evaluated dietary administration of 5 g/L oligofructose (OF), either alone or in combination with 1 g/L 2′-FL, the OF group displayed increased recognition memory after 1 h, but not 48 h, whereas the OF + 2′-FL group displayed increased recognition memory after 48 h only (122). These results suggest that the combination of oligosaccharides specifically affected long-term recognition memory.

Results from rodent studies support effects of 2′-FL on cognitive development and a mechanism of action through the gut-brain axis. Oral administration of 2′-FL to rat pups during the suckling period enhanced LTP in both 6-week-old and 1-year-old rats. Although 2′-FL-supplemented and control animals performed similarly on behavioural tests after weaning (4–6 weeks), rats that had received 2′-FL performed significantly better in the novel object recognition and Y maze paradigms at 1 year of age (123). A study in adult rats also showed that dietary 2′-FL, but not fucose, enhanced LTP in the hippocampus. Vagotomy inhibited the effects of 2′-FL on LTP, indicating that the cognitive benefits of 2′-FL are mediated via the vagus nerve and the gut-brain axis (124).

To date, only a few studies have evaluated the effects of combinations of HMOs on cognitive outcomes in experimental animal models. In a comprehensive study, the diets of Göttingen minipigs were supplemented with different combinations of HMOs from 2 to 11 weeks of age: either 4 g/L of fucosylated and neutral HMOs (FN; comprised of 2′-FL, DFL, LNT, LNnT); 0.68 g/L sialylated HMOs (SL; 3′-SL, 6′-SL); or 4 g/L of the combination (FN + SL). The minipigs were evaluated on multiple cognitive tests during and after the HMO supplementation period at three time points corresponding to infancy, adolescence and adulthood. Out of all the supplementation groups, cognitive tests and time periods, the only significant improvement associated with HMO supplementation was for the SL group on reference memory during the adolescence period, as mentioned above (114). This study underscores that cognitive effects of HMOs may be specific to the type of HMO, the particular cognitive task, and the developmental timeframe. Another study that evaluated combinations of HMOs also reported distinct effects dependent on HMOs and timing. In this study, newborn piglets received control milk replacer formula or formula supplemented with either 6.5 g/L BMOS (GOS, 3′-SL, 6′-SL), 1.5 g/L of HMOs (2′-FL, LNnT), or the combination of BMOS and HMOs. The group receiving HMOs displayed recognition memory after 1 h (125). Interestingly, in contrast to other studies where supplementation with 2′-FL, 3′-SL or 6′-SL individually did not enhance recognition memory at 48 h (116, 121), in this study, the group receiving the combination of BMOs and HMOs displayed recognition memory after 48 h (125). However, since the doses of the different HMOs varied considerably between and within studies (because they were intended to reflect clinically tested levels in infant formula), it is difficult to discern whether the effect might be a result of increased number and/or increased total dose of HMOs.

Collectively, the results from experimental animal studies indicate that 2′-FL, 3′-SL and 6′-SL may influence specific aspects of cognitive development through distinct mechanisms. However, these experimental studies also provide several examples where HMOs do not affect cognitive outcomes. In addition to the fact that the type and timing of a cognitive test may influence whether a positive effect is observed, there are several factors that limit the translation of animal models to human infants. First, milk oligosaccharide composition and gut microbiota composition are different in pigs and mice as compared to humans, with pigs and mice being deficient in fucosylated milk oligosaccharides and bifidobacteria (126–129). Supplementation of piglets with BMOS and/or HMOs did modulate gut microbiota composition, but with increased relative abundances of *Bacteroides* and/or *Blautia* (130), rather than of *Bifidobacterium*, as is typical in human infants. Since microbial metabolism of HMOs appears to be an important mechanism for their effects on cognitive development, particularly for the fucosylated HMOs, and the gut microbiota of pigs and mice did not evolve to utilize HMOs, negative results of cognitive studies should be interpreted with caution. Second, the cognitive demands of human infants are quite different from pigs and mice, for example with regards to language acquisition. Although animal models are an important tool for testing individual and combined HMOs in a controlled manner and for deciphering mechanisms, the roles of fucosylated and sialylated HMOs in cognitive development ultimately need to be substantiated by clinical interventional trials with manufactured HMOs.

## 3 Discussion

HMOs naturally occur as a complex mixture of molecules in breastmilk. By increasing the number of HMOs from one (2′-FL) or two (2′-FL and LNnT) to the seven HMOs that are available from large-scale production today and have been clinically investigated (2′-FL, 3-FL, DFL, LNnT, LNT, 3′-SL, 6′-SL), representation of the total HMO fraction in mature breastmilk increases from approximately 20%–24% to 45% (4). Although HMOs share some common biological functions, such as the promotion of bifidobacteria, HMOs also have distinct structural differences and unique biological functions, as presented in the examples above. By combining structurally diverse HMOs composed of different monosaccharide building blocks (fucose, GlcNAc, sialic acid), a closer recapitulation of the composition of breastmilk is achieved and greater health benefits are expected, including increased beneficial gut bacteria, defense against a broader range of pathogens, and potentially enhanced cognitive development.

Current knowledge on the mechanisms and health benefits of HMOs is based mostly on research that has been conducted on the smaller HMO structures, particularly the tri- and tetrasaccharides, due to their availability. However, several of the most abundant HMOs in breastmilk are pentasaccharides such as the lacto-N-fucopentaoses (LNFP I-VI) and the sialyllacto-N-tetraoses (LSTa-c) and hexasaccharides such as the lacto-difucohexaoses (LNDFH-I-III) and disialyllacto-N-tetraose (DSLNT) (4). In the context of precision fermentation technology, these larger HMO structures pose certain technical challenges. The enzymes required to catalyze certain glycosidic linkages in larger HMO structures are rare or lacking in the bacterial domain. Production of larger HMOs involves more enzymatic steps, which may lead to a more complex set of side products, and larger HMOs may not be efficiently transported out of the production organism, which is necessary to achieve high yields. Hence, alternative approaches need to be developed and optimized to produce larger HMO structures.

Efforts to manufacture larger HMOs may be warranted, since some of the larger, more structurally complex HMOs may provide unique and important health benefits. For example, lower concentrations of DSLNT have been associated with development of necrotizing enterocolitis in preterm infants (131, 132), motivating additional research to examine whether supplementation with DSLNT could prevent this life-threatening disease. In addition, a large-scale screen of a human milk glycan library that evaluated binding of a human neonatal rotavirus strain revealed that the seven high-affinity binding targets were all hexasaccharides or larger structures (76). This example highlights that there are likely important and not yet discovered roles for larger and more complex HMOs, particularly in the ability of HMOs to bind pathogens and reduce the risk of infection, since binding interactions are very specific due to structural constraints.

Scientific knowledge and production capabilities for HMOs have made enormous progress since the first observations on the unique bacterial composition of breastfed infant stool and the carbohydrate fraction of human milk. Although fortification with HMOs brings infant formula compositionally closer to breastmilk, additional clinical data in infants is needed to confirm some of the health benefits supported only by preclinical data, such as beneficial immunomodulation or enhanced cognitive development. Since HMO exposure occurs during a critical window of development, the potential longer-term benefits of HMO supplementation during infancy on childhood health outcomes such as immune function (e.g., allergy) and metabolism (e.g., obesity) need to be evaluated. Much work remains to increase understanding on the redundant and differential benefits of specific HMOs, to evaluate the full range of HMO physiological effects in clinical trials, and to increase the number and availability of manufactured HMOs worldwide.
